# Arthropods as the Engine of Nutrient Cycling in Arid Ecosystems

**DOI:** 10.3390/insects12080726

**Published:** 2021-08-14

**Authors:** Nevo Sagi, Dror Hawlena

**Affiliations:** Risk-Management Ecology Lab, Department of Ecology, Evolution & Behavior, The Alexander Silberman Institute of Life Sciences, The Hebrew University of Jerusalem, Jerusalem 9190401, Israel; dror.hawlena@mail.huji.ac.il

**Keywords:** arthropods, burrow, desert, detritivores, litter decomposition, nutrient acquisition, zoogeochemistry

## Abstract

**Simple Summary:**

Nutrient cycling in terrestrial ecosystems requires moisture. Deserts are characterized by low precipitation and extreme surface temperature that limit biological activity. Attempts to resolve how nutrients recycle despite these constraints were focused primarily on abiotic factors. We suggest that macro-arthropods may play a disproportionally large role in resolving this conundrum. Macro-arthropods are prevalent in deserts and many of them are burrowing detritivores that can remain active during long dry periods. Desert arthropods process and transport plant litter nutrients belowground, where the conditions are favorable for decomposer activity. Consequently, arthropods may accelerate the recycling rate of plant litter nutrients to inorganic nutrients that become available for plant use. This generates a vertical nutrient recycling loop (VRL) that may also assist in explaining how desert plants receive nutrients when the shallow soil is dry. Macro-arthropods may also regulate the spatiotemporal distribution of nutrients by transporting them between patches and across ecosystems. Burrowing activity that alters the desert microtopography and reduces soil salinity may contribute further to creating hotspots of productivity and biological diversity in the otherwise poor desert environment. We conclude that macro-arthropods may play a key role in explaining how desert ecosystems function, and that better understanding of this unique role may assist combating desertification and restoring degraded arid lands.

**Abstract:**

Nutrient dynamics in most terrestrial ecosystems are regulated by moisture-dependent processes. In drylands, nutrient dynamics are often weakly associated with annual precipitation, suggesting that other factors are involved. In recent years, the majority of research on this topic focused on abiotic factors. We provide an arthropod-centric framework that aims to refocus research attention back on the fundamental role that macro-arthropods may play in regulating dryland nutrient dynamics. Macro-arthropods are prevalent in drylands and include many detritivores and burrowing taxa that remain active during long dry periods. Macro-arthropods consume and process large quantities of plant detritus and transport these nutrients to the decomposer haven within their climatically buffered and nutritionally enriched burrows. Consequently, arthropods may accelerate mineralization rates and generate a vertical nutrient recycling loop (VRL) that may assist in explaining the dryland decomposition conundrum, and how desert plants receive their nutrients when the shallow soil is dry. The burrowing activity of arthropods and the transportation of subterranean soil to the surface may alter the desert microtopography and promote desalinization, reducing resource leakage and enhancing productivity and species diversity. We conclude that these fundamental roles and the arthropods’ contribution to nutrient transportation and nitrogen fixation makes them key regulators of nutrient dynamics in drylands.

## 1. Introduction

Drylands constitute up to 45% of the Earth’s terrestrial surface [1] but in comparison to other biomes remain poorly understood. In most biomes nutrient cycling is regulated primarily by various moisture-dependent biological processes, such as microbial decomposition and roots nutrient acquisition, that occur mainly near the soil surface [2]. Drylands are characterized by low moisture levels, infrequent and unpredictable precipitation, and extreme surface temperatures. These conditions may limit moisture-dependent biological activity in the shallow soil to short pulses following rain events, decelerating nutrient cycling [3,4,5]. Nevertheless, field observations from drylands show only a modest correlation between rates of nutrient cycling and precipitation, suggesting that additional factors are involved [6].

For almost 50 years, ecologists have been struggling to solve this puzzle and identify what additional factors may assist in accelerating nutrient cycling in drylands [3,4,5]. Noy-Meir (1974) emphasized the potentially important role of “*macrodecomposition* by detritivoric arthropods”, but suggested that weathering and erosion by wind may also assist in accelerating desert nutrient cycling [4]. Meentemeyer (1978) echoed Noy Meir’s hypothesis, proposing that “when soil animals are excluded from a decomposing litter, there is insufficient fragmentation leading to much-reduced consumption by microorganisms” [7]. Whitford et al. (1981) later added that thermal and photodegradation and alternative water sources may also contribute to the observed pattern, but they too emphasized the foundational role of arthropods, especially termites [8], for which additional empirical support was later provided [9,10,11].

Moorhead and Reynolds (1989) marked a paradigm shift moving the focus from animals to abiotic factors. They claimed that “non-biological processes, such as the leaching of soluble and photochemical degradation of lignins, may account for much of the total litter loss” [12]. Austin and Vivanco (2006) provide another cornerstone in diverting research effort toward abiotic factors [13]. In this important paper and many of the studies that followed, the researchers used litter bags or cages covered with mesh that exclude macrofauna and prevent litter redistribution, to assess litter mass loss in the presence or absence of focal abiotic factors. These studies offer strong experimental evidence that photodegradation, thermal degradation, and alternative sources of moisture, such as fog, dew, and atmospheric water vapor, can assist in explaining the unexpectedly high decomposition rate of exposed plant litter [14,15,16]. However, these studies also unintentionally narrow the original question of how “a considerable proportion of litter and standing dead material of ephemeral plants has been observed to disappear even in dry periods” [4] to the much-reduced question of what abiotic factors accelerate the decomposition of exposed surface litter that is neither being transported and ingested by macrofauna nor being transported and mixed with soil by wind or water flow. Moreover, by focusing on abiotic factors, researchers may have missed the contribution of macro-arthropods to other poorly understood processes of desert nutrient dynamics such as desalinization, acquisition of nutrients by plants, nutrients spatial distribution, or nitrogen fixation. Here, we provide a general framework that examines how macro-arthropods may assist resolving longstanding questions of spatiotemporal nutrient dynamics in drylands. In doing so, we aim to rip the fine mesh of the litter bags and inspire researchers to refocus their attention back to the potentially important regulatory role that macro-arthropods play in arid ecosystems.

## 2. Macro-Arthropods in Deserts

Plant litter is the main resource for primary consumers in many deserts, due to the spatiotemporal scarcity of green plant material that limits herbivory. Therefore, the primary consumer guild in drylands tends to be dominated by detritivorous arthropods (e.g., termites, isopods, and tenebrionid beetles) [17]. These macro-detritivores are preyed upon mainly by small ectothermic arthropods (e.g., spiders, scorpions, and solifuges) and reptiles, hence constitute the base of many energetically efficient food chains [18].

Macro-arthropods show many adaptations to the harsh environmental conditions in drylands. These include morphological and physiological characters that minimize water loss such as impermeable cuticle, discontinuous ventilation cycle, and excretion of nitrogenous waste compounds that are insoluble in water such as guanine or uric acid [19]. Moreover, many desert arthropods evade the harsh climatic conditions by shifting to nocturnal activity, using mud galleries for aboveground activity, and sheltering for extended periods in burrows where they experience more favorable microclimatic conditions [19,20]. These adaptations allow macrofauna to remain active even during extended dry periods when mesofauna and microorganisms cannot.

Burrowing arthropods serve as key ecological engineers by regulating the physical and chemical conditions both above and belowground [21]. Consequently, macro-arthropods are expected to play a central role in desert nutrient cycling. In the following sections, we will review the different pathways by which macro-arthropods regulate nutrient cycling in drylands.

## 3. Regulation of Plant Litter Removal

Sporadic evidence from various drylands concurs with the longstanding but understudied hypothesis that macro-arthropods account for a significant portion of litter removal from the desert surface [4]. In the Chihuahuan desert, termites are responsible for 50% of the leaf litter removal [11,22,23], and in the hot and dry Baza basin detritivores increased plant litter mass loss by a factor of 1.23 [24]. In the Negev desert macro-detritivores accounted for 89% of the litter removal, and the highest removal rate was observed in the dry and hot summer when the contribution of microorganisms and mesofauna was negligible [25].

A comprehensive meta-analysis that explored the effect of soil fauna exclusion on litter decomposition questions these findings [26]. They found that exclusion of soil fauna reduces litter mass loss by 35% globally, but only by 18% when focusing on drylands. We believe that these findings do not reflect the actual regulatory role that macro-arthropods play in hot deserts. This is because evidence from both cold and hot deserts and of mesofauna and macrofauna were all pooled together. Arthropods are ectotherms and cannot remain active during long cold periods. Thus, macro-arthropods are expected to be less abundant and have a lesser effect on litter removal in cold deserts [27,28]. Moreover, mesofauna cannot remain active near the surface during dry periods, possibly confounding the results of the analysis [29]. As of now, not enough data are available to explicitly evaluate the unique role that macro-detritivorous arthropods play in regulating plant litter removal in hot drylands. Future research should explore this question across different drylands by using litter baskets that exclude or enable macro-arthropod entry.

## 4. Regulation of Plant Litter Decomposition via Ingestion and Fragmentation

The fate of the litter removed by arthropods varies greatly. A large portion of the removed litter is being transferred unconsumed for later use. This fragmented litter allows higher microbial colonization and faster decomposition rate due to larger surface area than intact litter [30]. The ingested litter is either being assimilated into the arthropod body and used for secondary production or being egested as fecal matter. Part of the assimilated nutrients is excreted back to the environment in readily available forms such as CO_2_, ammonia, uric acid, guanine, or phosphate, accelerating the cycling of plant nutrients. The ingested litter that was not assimilated is released back to the environment as highly fragmented and partly decomposed fecal pellets [30]. Feces are expected to decompose faster than unprocessed plant litter [31] but see a detailed discussion in [32]. In a recent study, Joly et al. (2020) used 36 combinations of taxonomically different macro-detritivores and litter of various plant species and found that across litter and detritivore species, litter conversion into detritivore feces enhanced surface area and organic matter lability and thereby accelerated carbon cycling. Notably, the positive conversion effect on feces quality and decomposition increased with decreasing quality and decomposition of intact litter [33]. Thus, transportation and ingestion of plant litter by detritivorous arthropods is expected to accelerate nutrient cycling. This effect is expected to be exceptionally important in drylands due to the high detritivore abundance, the low nutritional quality of plant materials that characterize these ecosystems, and the very limited surface activity of microbes and mesofauna [34].

## 5. Regulation of Plant Litter Decomposition via Engineering Effects

Burrowing detritivores are the main vector of litter nutrients transfer in the forms of fragmented plant litter, feces, and labile excretions from the surface to the belowground realm [35,36]. Animal burrows promote the mineralization of these nutrients via the intertwined effects of climatic and nutrients facilitation. Burrows enhance water infiltration and soil aeration, and buffer the thermal extremes, creating a more favorable microclimate for microbes and mesofauna [37,38,39,40,41,42,43]. For example, in mesic systems burrows of earthworms and termites improve soil macro-porosity by 20–100 percent [44,45]. In soils of the Australian arid zones, ant nests quadruple water infiltration rate [37] and lizard burrow systems provide thermal buffering of up to 40 °C difference from surface temperatures [46]. These effects are expected to be most pronounced in drylands where soil surface is often covered with biological soil crust that limits water infiltration and aeration, and rapidly dries up following precipitation events. In the Negev desert, the climatic facilitation in arthropod burrows enables prolonged mineralization episodes after rain events that substantially alter the typical pulse dynamics of litter decomposition in drylands [47]. This effect leads to twofold higher litter mass loss in artificial holes that mimic just the climatic facilitation of animal burrows than aboveground [47].

The extended activity of animals within burrows enriches the burrow environment with labile nutrients [48,49,50,51]. For example, Sagi et al. (2019) found a 1.5-fold increase in ammonium, a twofold increase in nitrate, and a 1.3-fold increase in phosphate concentrations near the vertical burrow of desert isopods (*Hemilepistus reaumuri*) compared with 20 cm away from the burrow. Notably, nutrient enrichment was not limited to the burrow walls but extended up to a distance of about 10 cm from the vertical burrows [25]. In another example, Nutting et al. (1987) measured an 11-fold increase in ammonium and a twofold increase in phosphate in soil brought to the surface by Sonoran desert termites compared to adjacent surface soil [49]. Consequently, animal burrows provide also essential resources that facilitate microbial and meso-decomposer activity. This resource facilitation may lead, for example, to higher plant litter mass loss in nutritionally enriched burrows of desert isopods compared to the same plant litter in artificial holes of similar dimensions [47].

The integrative effect of the climatic and nutrient facilitation provides favorable conditions for the activity of microorganisms and mesofauna, accelerating the mineralization of litter-derived nutrients (i.e., fecal pellets and litter residues) within the burrow [47]. This potentially important pathway of litter decomposition highlights the need to shift research attention from focusing on abiotic factors that assist in explaining decomposition rates on the dry desert surface to biotic vectors that remove surface litter to the microbial havens belowground.

## 6. Desert Arthropods and the Vertical Nutrient Recycling Loop (VRL)

Detritivore burrows may serve as conduits of nutrients into deeper soil layers, altering the vertical distribution of nutrients in the oligotrophic desert soil [15]. Nutrients distribution in the soil profile is largely regulated by nutrient deposition (e.g., weathering and atmospheric deposition), nutrient leaching, and biological cycling that include acquisition of inorganic nutrients by plants and litter mineralization. Leaching moves nutrients downward and may increase nutrient concentrations with depth. In contrast, biological cycling generally moves nutrients upwards. This is because a large proportion of the nutrients absorbed by plants are transported aboveground and then recycled near the surface, producing nutrient distributions that are shallower or decrease with depth [52,53]. Since a large portion of the plant nutrients in arid lands are expected to be transported and mineralized belowground by animals, the biological cycling in these ecosystems may play a different role, generating nutrient concentrations that increase rather than decrease with the burrow depth.

**Figure 1:**
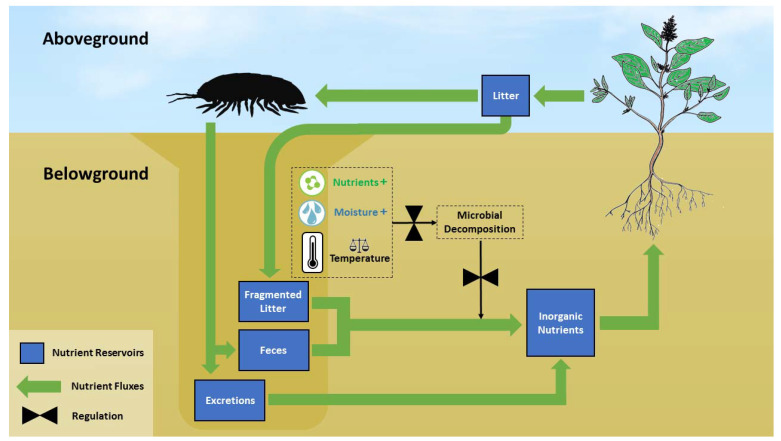
The vertical nutrient recycling loop (VRL) hypothesis: detritivorous macro-arthropods account for a major part of litter removal from the desert surface. Fragmented litter, fecal pellets, and excreted nutrients accumulate within and around arthropod burrows. Mineralization of organic matter within the burrow is enhanced due to climatic and nutrient facilitation, resulting in further soil fertilization. Desert plants utilize the moister deep soil to acquire nutrients from enriched patches around arthropod burrows, completing a vertical rather than a shallow nutrient loop.

The fate of the dense subterranean nutrient patches near macro-arthropod burrows is largely unknown. These nutrients may remain buried for extended periods, exiting contemporary nutrient cycling. However, more likely is that these nutrients may be used by desert plants, completing a vertical nutrient recycling loop (hereafter VRL; Figure 1). This largely hypothetical VRL may assist in explaining how desert perennials acquire nutrients in the moisture-deprived environment of the desert surface.

The episodic nature of water availability on the desert surface challenges the ability of perennial plants to take up nutrients from the surrounding soil. In most ecosystems plant roots acquire nutrients from the topsoil because most mineralization processes tend to occur there, creating a rich nutrient layer [54]. In deserts, plants had to survive during long periods of dry shallow soil, and brief and unpredicted wet episodes. To solve this challenge, some desert perennials produce fast-growing ephemeral roots following wetting events, allowing quick capture of nutrients as long as soil moisture holds [55]. Symbiotic interactions with drought-resistant fungi and other organisms within the biological soil crust can also facilitate rapid nutrient acquisition near the desert surface [5].

Plants may forage in deep soil for buried nutrients [56]. Many desert plants develop deep and extensive root systems in search of water [57,58]. Given that deep soil remains moist for extended periods, this may allow desert plants to continue acquiring buried nutrients when the topsoil dries. Studies from various ecosystems demonstrated that plant roots can forage selectively near animal burrows [59], and that bioturbation may lead to changes in rooting depth and root architecture [60,61], with positive consequences to plant biomass and nutritional condition [50,60,62]. For instance, in a minirhizotron experiment, the roots of *Achillea millefolium* occurred more frequently in earthworm (*Lumbricus terrestris* L.) burrows than in soil matrix or even soil cracks [59]. We propose that the arthropod-driven VRL is key for understanding nutrient cycling and plant nutrient acquisition in drylands. However, the importance and generality of this hypothetical VRL are yet to be determined.

## 7. Regulation of Nutrient Spatial Distribution within Ecosystems

Macro-arthropods can dramatically affect the spatial distribution of nutrients in drylands. Many arid ecosystems consist of two patch types: a matrix of crusted soil characterized by microphytic communities, and distinct patches of perennial shrubs with a mound and herbaceous understory (macrophytic patches). The microphytic patches that consist of algae, cyanobacteria, lichens, and mosses, creates a nutritionally poor and relatively impermeable soil crust [63,64,65,66]. The macrophytic patches, on the other hand, enable increased water infiltration and resource accumulation and serve as a refuge for seeds and seedling. Consequently, the macrophytic patches constitute “islands of fertility” in the otherwise nutritionally poor environment [66].

**Figure 2:**
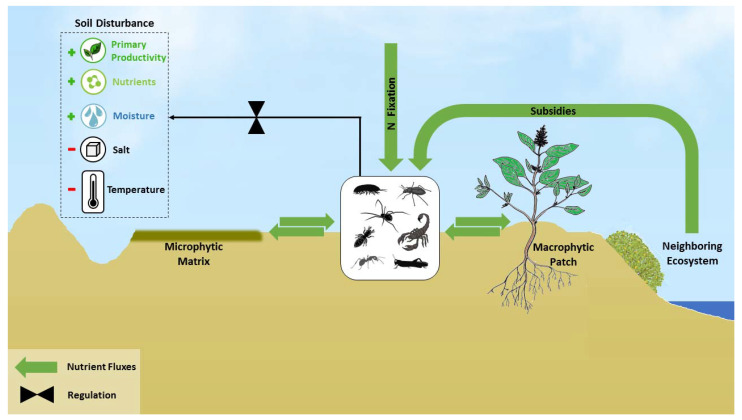
Pathways by which arthropods affect desert nutrient dynamics: arthropods transport nutrients between macrophytic patches and microphytic matrix, import nutrients from more productive neighboring ecosystems, and create soil disturbances that accumulate water and nutrients and provide favorable abiotic conditions for primary producers. Arthropods hosting diazotrophic bacteria in their gut can fix atmospheric N, resulting in soil N enrichment and enhanced N cycling.

Arthropod activity can significantly disturb this distinct spatial mosaic by transporting nutrients between the two patch types (Figure 2). In many deserts, animals cannot construct stable burrows in the loose organic-rich soil beneath shrub canopies [67,68]. Thus, many macro-arthropods transport nutrients from the macrophytic patches to their burrows, creating a nutrient-rich patch around the burrow’s entrance [25,49,69]. Consequently, macro-arthropod activity may create a more complex spatial mosaic of nutrient distribution [70,71], or blur the distinct differences in nutrient concentration between patch types. For instance, desert isopods evacuate soil, fecal pellets, and uneaten detritus from their burrow and mound them around the entrance. These loose mounds are much richer in nutrients, with a 1.5-fold increase in ammonium and a 9.8-fold increase in nitrate compared with adjacent soil crust [25]. In the Chihuahuan desert, the large differences in soil nitrogen between shrub patches and interspaces were canceled by termite activity [72]. This is possibly due to the deposition of nitrogen in the termite foraging galleries that crisscrossed the interspaces [49,51].

Drylands with an unstable surface such as sand dunes may stimulate a different pattern. In such ecosystems, burrowing activity tends to concentrate primarily within macrophytic patches where plant roots stabilize the otherwise loose substrate [73,74]. This may result in arthropods adding nutrients to the macrophytic patches, amplifying even further the differences in nutrient concentrations between the two patch types [75]. Shrub-dwelling sit-and-wait predators such as web-spiders may also amplify the contrast in nutrient concentration between macrophytic and microphytic patches. For instance, female white widow spiders (*Latrodectus pallidus*) in the Negev desert build their nests on shrubs where they capture foraging prey, consequently adding nitrogen to the patch and accelerating litter cycling within it (G. Szamet, unpublished data). We hypothesize that macro-arthropods play a key role in determining the spatial distribution of nutrients in drylands.

## 8. Regulation of Nutrient Spatial Distribution between Ecosystems

Macro-arthropods may serve as nutrient vectors also between neighboring ecosystems that may differ substantially in productivity (Figure 2). For example, in the hyper-arid Peruvian coastal desert, solifuges and scorpions prey on algae-feeding crustaceans on the pacific shore. They acquire 80–100% and 16–64% of their C, respectively, from this intertidal source [76]. Similarly, aquatic insects emerging from a Sonoran desert stream support an abundant and diverse spider community, accounting for 25% and 39% of N obtained by ground-active and orb-weaving spiders, respectively [77]. These nutrient subsidies may find their way into the soil in the form of predator waste and carcasses, ultimately fertilizing the desert ecosystem. We suggest that macro-arthropods in drylands play a key role in regulating the spatial distribution of nutrients both within and between ecosystems, and consequently control primary production and plant communities.

## 9. Soil Desalination via Burrowing Activity

Soil salinization occurs when evaporation exceeds precipitation and salts are not leached but remain in the upper soil layers in low-lying areas. Soil salinization decreases soil fertility and is considered a major process of land degradation in the world’s drylands [78]. Burrowing arthropods transfer subterranean saline soil to the surface. This easily erodible soil is later washed by run-off water and deposited downslope, arresting the salinization process (Figure 2). For instance, isopods in the Negev desert serve as desalination agents by transferring subterranean saline soil to the surface in the form of fecal pellets, turning the rocky slopes more favorable for both plants and animal activity [25,79]. Another example comes from a Caspian lowland semidesert, where animal burrowing activity resulted in desalination of the topsoil (0–50 cm depth) and salination of the deeper soil (50–100 cm) [80]. We hypothesize that macro-arthropods in drylands serve as key desalinization agents that indirectly regulate the plant community structure and ecosystem productivity.

## 10. Regulation of Primary Production via Soil Disturbances

Macro-arthropods disturb the microtopography of the soil surface during burrowing, egg-laying, and foraging activity, creating pits and mounds of subterranean soil. Such local disturbances often enable the accumulation of detritus [81], seeds [82], and runoff water [69,83], and alter the microclimatic conditions within the pits [84] (Figure 2). The accumulation of resources and the more favorable climatic conditions promote primary production and plant recruitment [85]. For example, ant nests in the Chihuahuan desert enhance soil moisture and nitrogen content and serve as hot spots for annual plants [85]. By altering the microtopography of the desert surface, animals may promote plant recruitment and reduce resource leakage enhancing the ecosystem productivity [86].

The occurrence of loose subterranean soil on the surface may promote plant diversity by increasing the spatial heterogeneity of soil physicochemical properties. For instance, piles of seedless and saline subterranean soil disposed of by scorpions assist the establishment of the competitive inferior annual *Salsola inermis* and probably eases its competition with the non-halophytes typically growing on the washed slopes of the Judean desert [87]. The creation of such distinct patches by macro-arthropods may promote habitat heterogeneity and species diversity.

## 11. Regulation of Nitrogen Fixation

Arthropods may also play an important role in regulating N inputs to arid ecosystems via N fixation. The presence of bacteria capable of fixing atmospheric N (diazotrophs) in arthropods was first discovered about 50 years ago in the guts of termites [88,89]. Since then, N-fixing bacteria were also found in Blattodea, Coleoptera, Diptera, Hemiptera, Hymenoptera, Lepidoptera, and Thysanoptera, and also in Diplopoda [90,91]. Unsurprisingly, N fixation is especially prevalent in arthropods that rely on N-poor resources [90]. High N resorption efficiency in desert plants leaves the remaining litter N locked up in recalcitrant compounds [92]. Therefore, fixation of atmospheric N might be a highly beneficial strategy for desert primary consumers, as a way to bypass this barrier [93]. In Australian arid zones, N fixation by termite gut symbionts is a major source of N in unvegetated patches [94] and of nitrate in groundwater [95]. In the Chihuahuan desert, termites (*Gnathamitermes tubiformans*) contribute about 66 g of nitrogen fixed per hectare per year [51]. In the Negev desert, N fixed in the gut of the root dwelling weevil *Conorhynchus pistor* is utilized by the annual plant *Salsola inermis* in a symbiotic interaction [96], with positive effects on root N content and seed mass [97]. Little is known about the importance of N fixation by arthropods to the overall N budget. We hypothesize that this understudied pathway of N addition may be especially important in drylands where other biotic pathways of N fixation may be moisture limited [91] (Figure 2).

## 12. Conclusions

Our understanding of nutrient dynamics in terrestrial systems relies heavily on studies from mesic systems. Attempts to apply this understanding to water-limited systems were proven insufficient. To bridge this conceptual discrepancy, ecologists put effort predominantly in revealing whether abiotic factors in the harsh desert surface may partially compensate for the lack of water. We provide a fresh view by suggesting that trophic and engineering activities of macro-arthropods may assist in explaining the spatiotemporal nutrient dynamics in drylands. We suggest that arthropods process large quantities of detritus on the desert surface and remove it belowground where the conditions are favorable for decomposers, and from where the already mineralized nutrients can be acquired by plants even when moisture in the shallow soil is scarce. This vertical nutrient recycling loop (VRL) may therefore be key for resolving the dryland decomposition conundrum (DDC), refocusing the attention back to macro-detritivores as suggested by Noy-Meir almost 50 years ago [4].

Arthropod transportation of nutrients between distinct patches both within and between ecosystems, and N fixation by arthropods’ gut symbionts may further assist in explaining the nutrient spatiotemporal distribution. We also suggest that by transporting subterranean soil aboveground and by altering the surface microtopography, arthropods can reduce resource leakage, enhance plant recruitment, and serve as vectors of desalination. Our arthropod-centric framework therefore suggests that rather than struggling to explain how biotic processes such as decomposition and N fixation occur when moisture is lacking, future studies should explore how arthropods provide favorable conditions for both producers and decomposers to function, and what are the ecosystem implications of these arthropod-controlled pathways. We hope that this line of research will contribute to the seemingly important link between biodiversity and ecosystem function, opening the door to new exciting research on how arthropods may assist in preventing desertification, and how we can use arthropods to help restore degraded, water-limited ecosystems.

## Data Availability

No new data were created or analyzed in this study. Data sharing is not applicable to this article.

## References

[1] Prăvălie R. (2016). Drylands extent and environmental issues. A global approach. Earth-Sci. Rev..

[2] Schlesinger W.H., Bernhardt E.S. (2013). Biogeochemistry.

[3] Noy-Meir I. (1973). Desert Ecosystems: Environment and Producers. Annu. Rev. Ecol. Syst..

[4] Noy-Meir I. (1974). Desert ecosystems: Higher trophic levels. Annu. Rev. Ecol. Syst..

[5] Collins S.L., Sinsabaugh R.L., Crenshaw C., Green L., Porras-Alfaro A., Stursova M., Zeglin L.H. (2008). Pulse dynamics and microbial processes in aridland ecosystems. J. Ecol..

[6] Austin A.T. (2011). Has water limited our imagination for aridland biogeochemistry. Trends Ecol. Evol..

[7] Meentemeyer V. (1978). Macroclimate and lignin controls of litter decomposition rates. Ecology.

[8] Whitford W.G. (1981). Exceptions to the AET model: Deserts and clear-cut forest. Ecology.

[9] Fowler H.G., Whitford W.G. (1980). Termites, microarthropods and the decomposition of the senescent and fresh creosotebush (*Larrea tridentata*) leaf litter. J. Arid Environ..

[10] MacKay W.P., Silva S.I., Loring S.J., Whitford W.G. (1987). The role of subterranean termites in the decomposition of above ground creosotebush litter. Sociobiology.

[11] Silva S.I., MacKay W.P., Whitford W.G. (1985). The relative contributions of termites and microarthropods to fluff grass litter disappearance in the Chihuahuan Desert. Oecologia.

[12] Moorhead D.L., Reynolds J.F. (1989). Mechanisms of surface litter mass loss in the northern Chihuahuan Desert: A reinterpretation. J. Arid Environ..

[13] Austin A.T., Vivanco L. (2006). Plant litter decomposition in a semi-arid ecosystem controlled by photodegradation. Nature.

[14] Adair E.C., Parton W.J., King J.Y., Brandt L.A., Lin Y. (2017). Accounting for photodegradation dramatically improves prediction of carbon losses in dryland systems. Ecosphere.

[15] Evans S., Todd-Brown K.E.O., Jacobson K., Jacobson P. (2020). Non-rainfall moisture: A key driver of microbial respiration from standing litter in arid, semiarid, and mesic Grasslands. Ecosystems.

[16] Day T.A., Bliss M.S., Placek S.K., Tomes A.R., Guénon R. (2019). Thermal abiotic emission of CO_2_ and CH_4_ from leaf litter and its significance in a photodegradation assessment. Ecosphere.

[17] Ayal Y., Polis G.A., Lubin Y., Goldberg D., Shachack M., Gosz J.R., Perevolotsky A., Pickett S.T.A. (2005). How can high animal diversity be supported in low-productivity deserts? The role of macrodetritivory and habitat physiognomy. Biodiversity in Drylands: Towards a Unified Framework.

[18] Ayal Y. (2007). Trophic structure and the role of predation in shaping hot desert communities. J. Arid Environ..

[19] Ward D., Ward D. (2009). Morphological, physiological, and behavioural adaptations of desert animals to the abiotic environment. The Biology of Deserts.

[20] Whitford W.G. (2002). Adaptations. Ecology of Desert Systems.

[21] Raza M.B., Bhoi T.K., Samal I. (2019). Soil arthropods as a nutrient enhancer. Int. J. Chem. Stud..

[22] Mackay W.P., Loring S.J., Zak J.C., Silva S.I., Fisher F.M., Whitford W.G. (1994). Factors affecting loss in mass of creosotebush leaf-litter on the soil surface in the northern Chihuahuan Desert. Southwest. Nat..

[23] Whitford W.G., Steinberger Y., Ettershank G. (1982). Contributions of subterranean termites to the “economy” of Chihuahuan Desert ecosystems. Oecologia.

[24] Megías A.G., Sánchez-Piñero F., Hódar J.A. (2011). Trophic interactions in an arid ecosystem: From decomposers to top-predators. J. Arid Environ..

[25] Sagi N., Grünzweig J.M., Hawlena D. (2019). Burrowing detritivores regulate nutrient cycling in a desert ecosystem. Proc. R. Soc. B Biol. Sci..

[26] García-Palacios P., Maestre F.T., Kattge J., Wall D.H. (2013). Climate and litter quality differently modulate the effects of soil fauna on litter decomposition across biomes. Ecol. Lett..

[27] van Geffen K.G., Berg M.P., Aerts R. (2011). Potential macro-detritivore range expansion into the subarctic stimulates litter decomposition: A new positive feedback mechanism to climate change?. Oecologia.

[28] Araujo P.I., Yahdjian L., Austin A.T. (2012). Do soil organisms affect aboveground litter decomposition in the semiarid Patagonian steppe, Argentina?. Oecologia.

[29] Santos P.F., Whitford W.G. (1981). The effects of microarthropods on litter decomposition in a Chihuahuan Desert ecosystem. Ecology.

[30] Frouz J. (2018). Effects of soil macro- and mesofauna on litter decomposition and soil organic matter stabilization. Geoderma.

[31] Joly F.X., Coq S., Coulis M., Nahmani J., Hättenschwiler S. (2018). Litter conversion into detritivore faeces reshuffles the quality control over C and N dynamics during decomposition. Funct. Ecol..

[32] David J.F. (2014). The role of litter-feeding macroarthropods in decomposition processes: A reappraisal of common views. Soil Biol. Biochem..

[33] Joly F.X., Coq S., Coulis M., David J.F., Hättenschwiler S., Mueller C.W., Prater I., Subke J.A. (2020). Detritivore conversion of litter into faeces accelerates organic matter turnover. Commun. Biol..

[34] Whitford W.G., Whitford W.G. (2002). Decomposition and nutrient cycling. Ecology of Desert Systems.

[35] Anderson J.M. (1988). Invertebrate-mediated transport processes in soils. Agric. Ecosyst. Environ..

[36] Jouquet P., Dauber J., Lagerlöf J., Lavelle P., Lepage M. (2006). Soil invertebrates as ecosystem engineers: Intended and accidental effects on soil and feedback loops. Appl. Soil Ecol..

[37] Eldridge D.J. (1993). Effect of ants on sandy soils in semi-arid eastern australia: Local distribution of nest entrances and their effect on infiltration of water. Aust. J. Soil Res..

[38] Laundre J.W. (1993). Effects of small mammal burrows on water infiltration in a cool desert environment. Oecologia.

[39] Phillips J.D. (2007). Development of texture contrast soils by a combination of bioturbation and translocation. Catena.

[40] Butler D.R., Butler W.D. (2009). The geomorphic effects of gophers on soil characteristics and sediment compaction: A case study from alpine treeline, Sangre de Cristo mountains, Colorado, USA. Open Geol. J..

[41] Coombes M.A., Viles H.A. (2015). Population-level zoogeomorphology: The case of the Eurasian badger (*Meles meles* L.). Phys. Geogr..

[42] Kinlaw A. (1999). A review of burrowing by semi-fossorial vertebrates in arid environments. J. Arid Environ..

[43] Whittington-Jones G.M., Bernard R.T.F., Parker D.M. (2011). Aardvark burrows: A potential resource for animals in arid and semi-arid environments. Afr. Zool..

[44] Edwards C.A., Bohlen P.J. (1996). Biology and Ecology of Earthworms.

[45] Gullan P.J., Cranston P.S. (2014). The Insects: An Outline of Entomology.

[46] Moore D., Stow A., Kearney M.R. (2018). Under the weather?—The direct effects of climate warming on a threatened desert lizard are mediated by their activity phase and burrow system. J. Anim. Ecol..

[47] Sagi N., Zaguri M., Hawlena D. (2021). Macro-detritivores assist resolving the Dryland Decomposition Conundrum by engineering an underworld heaven for decomposers. Ecosystems.

[48] Groffman P.M., Bohlen P.J., Fisk M.C., Fahey T.J. (2004). Exotic earthworm invasion and microbial biomass in temperate forest soils. Ecosystems.

[49] Nutting W.L., Haverty M.I., Lafage J.P. (1987). Physical and chemical alteration of soil by two subterranean termite species in Sonoran Desert grassland. J. Arid Environ..

[50] Evans T.A., Dawes T.Z., Ward P.R., Lo N. (2011). Ants and termites increase crop yield in a dry climate. Nat. Commun..

[51] Schaefer D.A., Whitford W.G. (1981). Nutrient cycling by the subterranean termite *Gnathamitermes tubiformans* in a Chihuahuan Desert ecosystem. Oecologia.

[52] Trudgill S.T. (1988). Soil and Vegetation Systems.

[53] Stark J.M., Caldwell M.M., Pearcy R. (1994). Causes of soil nutrient heterogeneity at different scales. Exploitation of Environmental Heterogeneity by Plants: Ecophysiological Processes above and below Ground.

[54] Hodge A., Lüttge U., Beyschlag W., Büdel B., Francis D. (2010). Roots: The acquisition of water and nutrients from the heterogeneous soil environment. Progress in Botany 71.

[55] Liu B., He J., Zeng F., Lei J., Arndt S.K. (2016). Life span and structure of ephemeral root modules of different functional groups from a desert system. New Phytol..

[56] McCulley R.L., Jobbágy E.G., Pockman W.T., Jackson R.B. (2004). Nutrient uptake as a contributing explanation for deep rooting in arid and semi-arid ecosystems. Oecologia.

[57] Schenk H.J., Jackson R.B. (2002). Rooting depths, lateral root spreads and below-ground/above-ground allometries of plants in water-limited ecosystems. J. Ecol..

[58] Schenk H.J., Jackson R.B. (2002). The global biogeography of roots. Ecol. Monogr..

[59] Cameron E.K., Cahill J.F., Bayne E.M. (2014). Root foraging influences plant growth responses to earthworm foraging. PLoS ONE.

[60] Daleo P., Iribarne O. (2009). The burrowing crab Neohelice granulata affects the root strategies of the cordgrass *Spartina densiflora* in SW Atlantic salt marshes. J. Exp. Mar. Biol. Ecol..

[61] Sasaki T., Kakinuma K., Yoshihara Y. (2013). Marmot disturbance drives trait variations among five dominant grasses in a Mongolian grassland. Rangel. Ecol. Manag..

[62] Villarreal D., Clark K.L., Branch L.C., Hierro J.L., Machicote M. (2008). Alteration of ecosystem structure by a burrowing herbivore, the plains vizcacha (*Lagostomus maximus*). J. Mammal..

[63] Jackson R.B., Caldwell M.M. (1993). Geostatistical patterns of soil heterogeneity around individual perennial plants. J. Ecol..

[64] Schlesinger W.H., Raikks J.A., Hartley A.E., Cross A.F. (1996). On the spatial pattern of soil nutrients in desert ecosystems. Ecology.

[65] Burke I.C., Lauenroth W.K., Vinton M.A., Hook P.B., Kelly R.H., Epstein H.E., Aguiar M.R., Robles M.D., Aguilera M.O., Murphy K.L. (1998). Plant-soil interactions in temperate grasslands. Biogeochemistry.

[66] Ochoa-Hueso R., Eldridge D.J., Delgado-Baquerizo M., Soliveres S., Bowker M.A., Gross N., Le Bagousse-Pinguet Y., Quero J.L., García-Gómez M., Valencia E. (2018). Soil fungal abundance and plant functional traits drive fertile island formation in global drylands. J. Ecol..

[67] Brown G., Scherber C., Ramos P., Ebrahim E.K. (2012). The effects of harvester ant (*Messor ebeninus* Forel) nests on vegetation and soil properties in a desert dwarf shrub community in north-eastern Arabia. Flora Morphol. Distrib. Funct. Ecol. Plants.

[68] Wagner D., Jones J.B. (2004). The contribution of harvester ant nests, *Pogonomyrmex rugosus* (Hymenoptera, Formicidae), to soil nutrient stocks and microbial biomass in the Mojave Desert. Environ. Entomol..

[69] James A.I., Eldridge D.J., Koen T.B., Whitford W.G. (2008). Landscape position moderates how ant nests affect hydrology and soil chemistry across a Chihuahuan Desert watershed. Landsc. Ecol..

[70] Baubin C., Farrell A.M., Šťovíček A., Ghazaryan L., Giladi I., Gillor O. (2019). Seasonal and spatial variability in total and active bacterial communities from desert soil. Pedobiologia.

[71] Jones J.B., Wagner D. (2006). Microhabitat-specific controls on soil respiration and denitrification in the Mojave Desert: The role of harvester ant nests and vegetation. West. N. Am. Nat..

[72] Brown M.F., Whitford W.G. (2003). The effects of termites and straw mulch on soil nitrogen in a creosotebush (*Larrea tridentata*) dominated Chihuahuan Desert ecosystem. J. Arid Environ..

[73] Li X.R., Gao Y.H., Su J.Q., Jia R.L., Zhang Z.S. (2014). Ants mediate soil water in arid desert ecosystems: Mitigating rainfall interception induced by biological soil crusts?. Appl. Soil Ecol..

[74] Henschel J.R., Lubin Y.D. (1997). A test of habitat selection at two spatial scales in a sit-and-wait predator: A web spider in the Namib Desert dunes. J. Anim. Ecol..

[75] Chen Y.W., Li X.R. (2012). Spatio-temporal distribution of nests and influence of ant (*Formica cunicularia* Lat.) activity on soil property and seed bank after revegetation in the Tengger Desert. Arid Land Res. Manag..

[76] Catenazzi A., Donnelly M.A. (2007). The Ulva connection: Marine algae subsidize terrestrial predators in coastal Peru. Oikos.

[77] Sanzone D.M., Meyer J.L., Marti E., Gardiner E.P., Tank J.L., Grimm N.B. (2003). Carbon and nitrogen transfer from a desert stream to riparian predators. Oecologia.

[78] Thomas D.S.G., Middleton N.J. (1993). Salinization: New perspectives on a major desertification issue. J. Arid Environ..

[79] Yair A. (1995). Short and long term effects of bioturbation on soil erosion, water resources and soil development in an arid environment. Geomorphology.

[80] Shabanova N.P., Lebedeva-Verba M.P., Bykov A.V. (2010). Morphological and chemical properties of the meadow-semidesert soil complexes of the Khaki playa (the Caspian Lowland) and the influence of the biogenic factor on them. Eurasian Soil Sci..

[81] Throop H.L., Belnap J. (2019). Connectivity dynamics in dryland litter cycles: Moving decomposition beyond spatial stasis. BioScience.

[82] Beattie A.J., Culver D.C. (1983). The nest chemistry of two seed-dispersing ant species. Oecologia.

[83] Elkins N.Z., Sabol G.V., Ward T.J., Whitford W.G. (1986). The influence of subterranean termites on the hydrological characteristics of a Chihuahuan Desert ecosystem. Oecologia.

[84] Gutterman Y. (1997). Spring and summer daily subsurface temperatures in three microhabitats in a flat natural loess area in the Negev Desert, Israel. J. Arid Environ..

[85] Eldridge D.J., Whitford W.G., Duval B.D. (2009). Animal disturbances promote shrub maintenance in a desertified grassland. J. Ecol..

[86] Shachak M., Brand S., Gutterman Y. (1991). Porcupine disturbances and vegetation pattern along a resource gradient in a desert. Oecologia.

[87] Danin A. (1994). Association of Salsola inermis and scorpion burrows in leached soils in the Judean Desert, Israel. Isr. J. Plant Sci..

[88] Benemann J.R. (1973). Nitrogen fixation in termites. Science.

[89] Breznak J.A., Brill W.J., Mertins J.W., Coppel H.C. (1973). Nitrogen fixation in termites. Nature.

[90] Bar-Shmuel N., Behar A., Segoli M. (2020). What do we know about biological nitrogen fixation in insects? Evidence and implications for the insect and the ecosystem. Insect Sci..

[91] Nardi J.B., Mackie R.I., Dawson J.O. (2002). Could microbial symbionts of arthropod guts contribute significantly to nitrogen fixation in terrestrial ecosystems?. J. Insect Physiol..

[92] Deng M., Liu L., Jiang L., Liu W., Wang X., Li S., Yang S., Wang B. (2018). Ecosystem scale trade-off in nitrogen acquisition pathways. Nat. Ecol. Evol..

[93] Meng F., Bar-Shmuel N., Shavit R., Behar A., Segoli M. (2019). Gut bacteria of weevils developing on plant roots under extreme desert conditions. BMC Microbiol..

[94] Cook G.D., Dawes-Gromadzki T.Z. (2005). Stable isotope signatures and landscape functioning in banded vegetation in arid-central Australia. Landsc. Ecol..

[95] Barnes C.J., Jacobson G., Smith G.D. (1992). The origin of high-nitrate ground waters in the Australian arid zone. J. Hydrol..

[96] Bar-Shmuel N., Rogovin E., Rachmilevitch S., Friedman A.L.L., Shelef O., Hoffmann I., Rosenberg T., Behar A., Shavit R., Meng F. (2018). Tripartite symbiosis of plant-weevil-bacteria is a widespread phenomenon in the Negev Desert. Sci. Rep..

[97] Shelef O., Helman Y., Friedman A.L.L., Behar A., Rachmilevitch S. (2013). Tri-party underground symbiosis between a weevil, bacteria and a desert plant. PLoS ONE.

